# A Manual for the Glasgow Outcome Scale-Extended Interview

**DOI:** 10.1089/neu.2020.7527

**Published:** 2021-08-13

**Authors:** Lindsay Wilson, Kim Boase, Lindsay D. Nelson, Nancy R. Temkin, Joseph T. Giacino, Amy J. Markowitz, Andrew Maas, David K. Menon, Graham Teasdale, Geoffrey T. Manley

**Affiliations:** ^1^ Division of Psychology, School of Natural Sciences, University of Stirling, Stirling, United Kingdom.; ^2^ Harborview Medical Center, Department of Neurological Surgery, University of Washington, Seattle, Washington, USA.; ^3^ Medical College of Wisconsin, Milwaukee, Wisconsin, USA.; ^4^ Spaulding Rehabilitation Hospital, Charlestown, Massachusetts, USA.; ^5^ Brain and Spinal Injury Center, University of California, San Francisco, San Francisco, California, USA.; ^6^ Department of Neurosurgery, Antwerp University Hospital and University of Antwerp, Edegem, Belgium.; ^7^ Division of Anaesthesia, University of Cambridge, Addenbrooke's Hospital, Cambridge, United Kingdom.; ^8^ Mental Health and Wellbeing in the Institute of Health and Wellbeing at the University of Glasgow Medical School, Glasgow, United Kingdom.

**Keywords:** clinical outcome assessment, Glasgow Outcome Scale-Extended, GOSE, traumatic brain injury

## Abstract

The Glasgow Outcome Scale-Extended (GOSE) has become one of the most widely used outcome instruments to assess global disability and recovery after traumatic brain injury. Achieving consistency in the application of the assessment remains a challenge, particularly in multi-center studies involving many assessors. We present a manual for the GOSE interview that is designed to support both single- and multi-center studies and promote inter-rater agreement. Many patients fall clearly into a particular category; however, patients may have outcomes that are on the borderline between adjacent categories, and cases can present other challenges for assessment. The Manual includes the general principles of assessment, advice on administering each section of the GOSE interview, and guidance on “borderline” and “difficult” cases. Finally, we discuss the properties of the GOSE, including strengths and limitations, and outline recommendations for assessor training, accreditation, and monitoring.

## Introduction

The Glasgow Outcome Scale (GOS) was published by Jennett and Bond^1^ in 1975 as an assessment of global outcome after severe brain injury. At the time that the GOS was developed, it was becoming increasingly well documented that traumatic brain injury (TBI) led to prolonged physical and mental consequences. The GOS was designed to capture how injury affected functioning in major areas of life. The original scoring was based on five possible categories of outcome (Table 1). Assessment involved using the authors' defining text as a guide when assigning an outcome category, and no record was made other than the final rating.

**Table 1.** Descriptions of the Categories of the Glasgow Outcome Scale

“1. Dead: As a direct result of brain trauma, or … due to secondary complications or other complications**”**
“2. Vegetative State: Patients who remain unresponsive and speechless….”
“3. Severe Disability: The patient is conscious but needs the assistance of another person for some activities of daily living every day.….”
“4. Moderate Disability: Such a patient is able to look after himself at home, to get out and about to the shops and to travel by public transport. However, some previous activities, either at work or in social life, are now no longer possible by reason of either physical or mental deficit….”
“5. Good Recovery: This indicates the capacity to resume normal occupational and social activities, although there may be minor physical or mental deficits…social outcome should be included in the assessment here, such as leisure activities and family relationships.”
Excerpted from Jennet and Bond (1975)^1^

The design of the GOS was innovative at the time and recognized two key points concerning the consequences of TBI. The first is that cognitive and mental health issues are an important cause of disability after brain injury. In the past, much of the follow-up after acute injury focused on physical problems, particularly the ability to walk; the GOS went beyond this relatively narrow approach. Second, the assessment demonstrated that global scales could be used to summarize outcome, eliminating the need to catalogue the wide varieties of impairment caused by injury. The consequences of impairment were captured by examining their end effect on major aspects of life after injury. The GOS joined the family of global outcome assessment scales that include the Rankin Scale^2^ used in stroke and the Karnovsky Performance Scale^3^ in cancer.

**FIG. 1.**
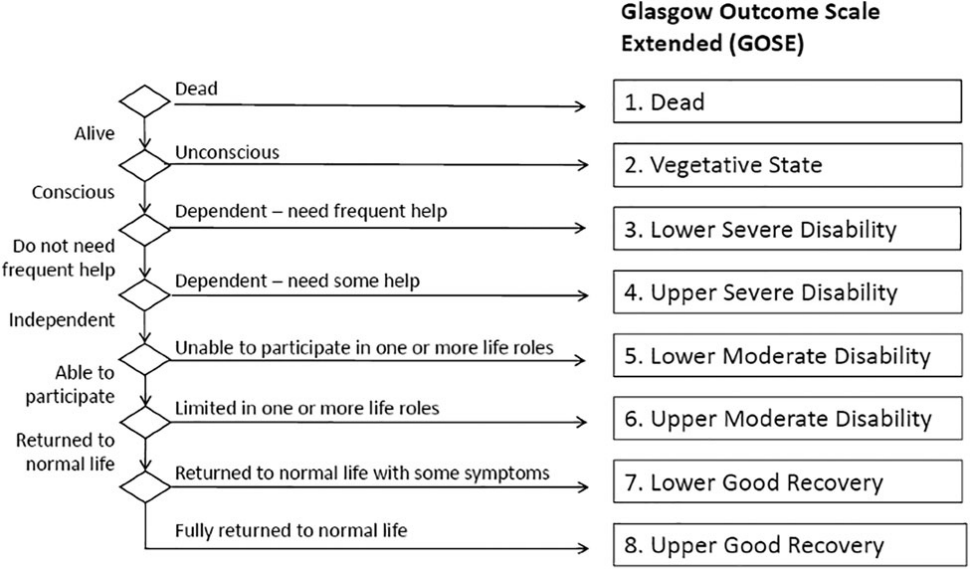
GOSE hierarchy of outcomes (adapted from Maas and colleagues^4^ with permission) GOSE, Glasgow Outcome Scale-Extended.

To increase the sensitivity of the GOS, Jennett and colleagues^5^ later suggested that categories of outcome could be divided into upper and lower bands to create an expanded 8-point scale. However, difficulties were documented in applying the GOS consistently, and the expanded version exacerbated this problem.^6^ Anderson and colleagues^7^ found that general practitioners were much more likely to rate patients as having a Good Recovery (GR) on the GOS than a psychologist who had carried out a neuropsychological assessment. In 1998, a structured version of the Glasgow Outcome Scale-Extended (GOSE) interview was published to help standardize procedures for scoring both the GOS and GOSE.^8^ The interview provides a set of guiding questions with which to assess the GOSE's domains or areas of functioning (the GOSE interview schedule; see Supplementary Materials, Appendix S1) and criteria for each category (Fig. 1; Table 2). The definitional rules for assessing outcome remain, but the interview questions allow the assessor to apply his or her judgment to resolve inconsistencies or probe for detail in the absence of information.

**Table 2.** Overview of the Criteria for the Categories of the GOSE

GOS 5-point scale	GOSE 8-point scale	Domain	Criteria
Dead	1. Dead		
Vegetative State	2. Vegetative State	Consciousness	
Severe Disability (SD) Conscious but dependent	3. Lower SD	Function in Home	Unable to look after themselves for 8 h
	4. Upper SD	Function in Home	Unable to look after themselves for 24 h OR
		Function Outside the Home	Unable to shop OR
			Unable to travel
Moderate Disability (MD) Independent but with limitations in one or more activities	5. Lower MD	Work/Study	Unable to work/study OR
		Social and Leisure Activities	Unable to participate OR
		Family and Friendships	Constant problems
	6. Upper MD	Work	Reduced work capacity OR
		Social and Leisure Activities	Participate much less OR
		Family and Friendships	Frequent problems
Good Recovery (GR) Return to normal life	7. Lower GR	Social and Leisure Activities	Participate a bit less OR
		Family and Friendships	Occasional problems OR
		Symptoms	Some symptoms affecting daily life
	8. Upper GR		No problems

GOSE, Glasgow Outcome Scale-Extended; GOS, Glasgow Outcome Scale.

The GOSE is atypical as a form of assessment given that it consists of a series of discrete categories arranged in a hierarchy (Fig. 1), and there is no sum score from individual items. In principle, the process of assigning a GOSE rating is simple: The choice points in the hierarchy are used to decide an outcome. The GOSE interview schedule is designed to facilitate this process by providing questions that elicit key information and by helping to define the borderlines between scored categories. For example, there are three questions concerned with the boundary between dependence and independence. Some cases are straightforward to classify. For example, a person who is conscious but needs a full-time caregiver would be scored in the Lower Severe Disability (SD) category; someone who is independent but unable to return to work or for students, their studies, is Lower Moderate Disability (MD); and a person who reports no problems or impairing symptoms is Upper GR. Experienced interviewers showed independent agreement on 78% of outcomes on the GOSE.^8^ However, there can be issues at borderlines and some cases that represent a challenge to assessment.

The GOSE focuses on change post-injury, but does not itself distinguish changes attributable to injury to the brain from disability caused, for example, by injury to other parts of the body. The GOSE can be used to assess the consequences of general trauma, including polytrauma, and in this case the effects of all kinds of injury are included. The decision about whether to assess the overall impact of injury or focus on the effects of brain injury will depend on the purpose of the study.

The GOSE has become widely adopted in TBI research studies. It has been embraced by regulators, including the U.S. Food and Drug Administration (FDA), as the primary clinical outcome assessment to prove efficacy in clinical trials in TBI.^9^ It is the only outcome recommended as “core” in the Common Data Elements for TBI.^10^ As part of the CENTER-TBI study, the interview has been translated into 17 languages. Achieving consistency in the application of the assessment remains a challenge, particularly in multi-center studies involving many assessors.

The aim of this article is to update and amplify guidance on the GOSE interview. The GOSE Manual, provided in its entirety following, was developed by investigators of the longitudinal, observational CENTER-TBI^11^ and TRACK-TBI^12^ studies to train assessors and thereby optimize reliable and reproducible GOSE outcome data. For an illustration of the Manual's practical application in the research setting, please see the accompanying article by Boase and colleagues.^13^ The manual does not seek to change the advice given in the original publication and keeps the original wording where appropriate,^8^ but it provides additional clarifications, queries, and examples based on our joint experience using the GOSE. In addition, we have tried to organize the points in a form that will be of practical use to assessors.

## The Glasgow Outcome Scale-Extended Manual

### Procedure overview

The questions do not need to be asked exactly given that they are written (see appendix for the GOSE interview schedule, Supplementary Materials, Appendix S1), but the central sense needs to be preserved. Some questions can be skipped depending on responses to previous questions. In sections where the person reports a limitation, the assessor questions further to confirm the disability.

### Summary of Glasgow Outcome Scale-Extended questions and administration steps

The steps in using the GOSE interview are as follows (further details are given in the *Notes to the interview* section and following):

- The interview begins with an introduction, for example: “I would like to ask you some questions about your daily life since the injury, and any problems that you have encountered.”
- Question 1 (Obey Commands): The question is generally relevant only for people who are very severely disabled. It will be skipped by the assessor if it is obvious that the person is able to communicate.
- Question 2 (Assistance at Home): Concerns independence in activities of daily living at home. If the person does need help, there are further questions about the kind of help that they need and how often they need it. If the person does not need assistance, then it is assumed that the answer to 2c concerning help before injury is also “No,” and the interview moves to Question 3.
- Questions 3 and 4 (Shopping and Travel): Focus on two key activities outside the home that characterize independent living in society.
- Question 5 (Work): Concerns the person's ability to resume employment and similar roles (e.g., for students, their academic pursuits). If the person did not participate before injury, this is recorded, and the other questions on work can be skipped.
- Question 6 (Social and Leisure Activities): Concerns how the person spends their free time. It is very unusual for someone not to have some engagement here pre-injury, but it may take questioning to establish what these activities were.
- Question 7 (Family and Friendships): Concerns problems arising in close relationships. There are some prompts here to help elicit whether the person has experienced mental/behavioral changes that impact relationships.
- Question 8 (Return to Normal Life): Covers symptoms that interfere with daily life. The assessor tries to ensure that the symptoms are a result of the injury, and also that they have an effect on daily living.

**Note for assessors**

When the person reports a problem/limitation, follow-up questions are asked. Complete the whole interview to check that answers provide a consistent picture.

### Assigning ratings on the Glasgow Outcome Scale-Extended

Overall rating: outcome categories are indicated against specific responses, and overall outcome is determined by the lowest outcome category indicated by the person's responses. The rating is only based on areas of function that have changed: that is, questions where there has been no change compared with pre-injury status are ignored for purposes of the rating.

The specific steps in scoring are:

1. Examine the responses and discount items where there has been no change (any difficulties experienced now are the same as before injury).
2. GOSE categories (vegetative state [VS] to upper GR) are shown in brackets beside specific responses.
3. The overall rating is the lowest outcome category indicated by the person's answers (after discounting limitations or problems before injury). Deaths are taken from records, and on the GOSE rating schema, “VS” or a score of 2 is the lowest category and “Upper GR” or a score of 8 is the highest. If the person has no limitations or impairing symptoms, then their GOSE rating is Upper GR.

**Note for assessors**

Working through the interview, the lowest outcome category is usually determined by the first section in which the person reports problems or limitations. Particular care is needed here when interviewing, given that the overall rating is determined by their responses.

### Notes to the interview

The following notes contain advice for assessors. Along with describing general rating principles, there is detailed information on interviewing, including follow-up questions, and advice on managing specific issues that can arise when interviewing.

### General principles

#### a. Use the best source of information available

In most cases, the patient is the best source, and their responses are taken at face value. However, unreliable reporting may be suggested by lack of realism in responses or answers that lack expected detail or are inconsistent. Information can be obtained from a person who is familiar with the daily routine of the patient, such as relatives or clinical staff. Eligible informants should be age ≥18 years and be in regular face-to-face contact with the patient (i.e., see them at least once a week). Always use the best source of information when assigning a rating.

**Note for assessors**

If in doubt about respondent reliability, obtain information from a surrogate. Multiple sources of information can be combined to determine an overall rating.

#### b. The rating depends on change from pre-injury function

The interview is concerned with identifying changes from pre-injury status; it is primarily changes that determine the outcome rating. Limitations before injury are not uncommon, and as far as possible the assessor tries to discount or ignore these in the overall rating. Questions are thus included concerning status before injury. The purpose of these questions is to confirm that the level of restriction represents a change with respect to the pre-trauma situation.

**Notes for assessors**

Sometimes it can be difficult to establish whether a person could do an activity before injury: In this case, it is easiest to ask the person what has changed since the injury.

“Current” status includes problems and limitations evident over the past week or so. If the person reports problems/limitations longer ago than this, then you may need to establish that these problems are still present.

#### c. Capability is considered as well as actual performance

The interview covers common activities and abilities, and in many instances simply finding out whether the person performs the activities is sufficient. However, sometimes it is necessary to judge whether the person is able to perform an activity, even if they do not engage in it in daily life. Financial or practical constraints, for example, might be a reason why a person does not engage in an activity, but this does not render the person disabled. On the other hand, the person would be considered disabled if the limitation is a result of physical or mental impairment from brain injury.

**Note for assessors**

You may need to ask follow-up questions to establish capability. Could they do the activity if it were really necessary? What is it that prevents performance?

#### d. Consistency between responses is important

The responses to the separate sections should generally be hierarchical (e.g., if a person indicates that they require assistance in the home, then it is potentially inconsistent if they answer that they go out alone for social and leisure activities). It would also be inconsistent if the person reported that they were unable to work, but also reported that they had no symptoms. Thus, responses to later questions may suggest revisions to earlier responses. The opportunity to check consistency is one of the reasons that it is important to complete the whole interview.

**Note for assessors**

If responses are inconsistent, you may need to go back to an earlier point of the interview to question further.

## Sections of the Interview

### Consciousness

**Q1: Is the** head-injured **person able to obey simple commands, or say any words?**

The first question is intended for patients who are alive but cannot be interviewed because they cannot respond to questions verbally or in writing. The rule of thumb that is used to identify patients in a VS (i.e., awake but with no sign of awareness) is whether or not they can obey commands or say any words. In practice, the question of whether the person is considered vegetative can usually be answered best by staff caring for the patient; if possible, a full assessment is recommended (see below).

**Skipping**

If the person is obviously more than minimally conscious, then Question 1 is skipped; start from Question 2. If the person is in a VS or minimally conscious state (MCS), then the rest of the interview is skipped.

#### Full assessment of patients in vegetative or minimally conscious state

A full assessment of patients in a VS or MCS is a specialized process requiring detailed tests of responsiveness. For example, the Coma Recovery Scale-Revised^14^ includes criteria for identifying patients in the VS, and these can be followed in deciding how the person is categorized on the GOSE. Visual pursuit (also known as “tracking”) is a specific aspect that has given rise to differences in classification in the past. Although the eyes may fleetingly turn to follow or fixate on an object, neither sustained visual fixation nor sustained pursuit are observed in a VS. For further details concerning the criteria for VS and MCS, please see Giacino and colleagues^15^ and Kondziella and colleagues^16^ Patients who fulfill criteria for MCS are rated as Lower SD.

**Specific issues**

Patients who do not obey commands, and this is considered to be attributable to language problems or severe cognitive impairment (e.g., dementia), are conscious and *should not* be rated as VS. Purposive behavior (reaching for food or grooming utensils, taking off articles of clothing, etc.) indicates consciousness in the absence of language.

### Independence in the home

**Q2a. Is the assistance of another person at home essential every day for some activities of daily living?**

Dependence here means that assistance is essential for the person on a daily basis. The types of activity that are relevant are those that are necessary for independence. Assistance may be essential when there is actual help (by another person) with an activity or there is a need for supervision, or the person needs prompting or reminding to do a task. Although dependency may be caused by physical impairment, after a TBI, the need for assistance often arises from mental changes alone.

**Skipping**

If the person does not need assistance, assume that they did not need help before (Q2c = no), and go to Question 3a (Shopping).

#### Follow-up questions

After asking the main question, the assessor can give the person examples (some are listed on the interview schedule; see Supplementary Materials, Appendix S1), to make clear the kinds of activities that are meant. The focus is on issues of safety and meeting needs in daily life: Will they get washed and dressed, and will they eat? Will they do this without prompting? Are they safe? (e.g., Are they at risk of burning the house down?); Can they handle small emergencies? (e.g., a glass is dropped and broken, a tap is left running causing a flood, a light goes out, it begins to get cold, or a stranger comes to the door). Establish that they could care for themselves, if necessary, for a 24-h period.

If help is needed, then ask: *What kinds of things do you get help with*? Sometimes assistance is reported for activities like taking a shower, or non-personal activities such as washing clothes, or household cleaning tasks. These do not count here, because they do not need to be done every day (e.g., showering) and thus are not essential for independence in daily life.

Establish whether they are incapable of the activity. Could they perform the activity if they really needed to? Could they manage on their own if need be? If they can, then assistance is not essential. Record responses based on the ability of the patient to perform the activity and not whether the patient actually performs the activity currently.

**Specific issues**

A difficulty may arise if an activity was not normally carried out before the injury. For example, someone may not usually prepare main meals for themself. In this case, it is sufficient that the person could, if the necessity arose, prepare food, even if this would only be a snack.

Many persons receive assistance in the sense of companionship or protection. The person may well benefit from this help, but such care does not mean that they are dependent in the sense required here. The need for supervision for safety reasons should be attributable to objective danger, rather than “just in case.”

Circumstances may mean that the person is never left alone. It is not necessary that the person is actually left alone, only that they *could* look after themselves if necessary. The stress here is thus not on being left alone, but on the ability to care for oneself.

Occasionally, people report needing daily assistance with a circumscribed activity, but they are otherwise independent in activities of daily living. Illustrative examples:

- A 60-year-old woman has a slight mobility problem with her left arm and is unable to put her hand behind her head. She wears her hair up, and now needs help each day to put her hair in place. She can use her right hand and arm normally. She has returned to work and to her usual social and leisure activities.
- A 73-year-old man gets help to wash his hair because he feels dizzy when raising and lowering his head, but otherwise is physically fit and can ride a bicycle.
- A 70-year-old man gets daily help with taking medication, but otherwise is independent outside the home and is back to his social and leisure activities.

In such cases, the assessor should not rate the person as SD, given that the isolated limitation is not consistent with the overall picture of independence.

**Q2b. Do they need frequent help or someone to be around at home most of the time?**

The patient is considered to be in the lower category of SD if they cannot be left for 8 h. This limit implies that a relative who is caring for them cannot work full time away from home.

**Q2c. Was assistance at home essential before the injury?**

Record whether the person was dependent for activities of daily life in the home before injury. If the person was disabled to the same extent before injury as they are now, record “yes.” If the person was dependent to some extent before injury, but their dependency has increased, record “no” to signify that there has been a change.

#### Follow-up questions

If it is necessary to establish a time limit, it can be helpful to ask what the maximum amount of time would be that they could look after themselves.

**Specific issues**

The central focus is on the person's ability to look after themself, rather than being left alone. The patient may never actually be on their own, but is nonetheless able to look after themself. The notes about companionship and safety issues also apply when considering whether the person can look after themself for 8 h.

### Independence outside the home

**Q3a. Are they able to shop without assistance?**

This refers to being able to buy items as part of daily living. Independence requires ability to plan, take care of money, and behave appropriately in public.

**Q3b. Were they able to shop without assistance before the injury?**

Record whether the person was able to shop independently before injury.

**Skipping**

If the person can now shop, assume that they could also do this before the injury (3b = yes), and go to Q4 (Travel).

#### Follow-up questions

If the person reports that they are unable to shop, then establish capability: *If your life depended on it, could you get out and buy even a single item? Can you go to a local shop to buy milk or bread*? If there is no local shop, you may need to ask the hypothetical question: *If there were a local shop, would you be able to buy something*?

**Specific issues**

The ability to shop does not mean carrying out a large shopping trip or being able to carry heavy items. It does not include online shopping given that this does not involve going outside the home.

**Q4a. Are they able to travel locally without assistance?**

This question refers to whether or not the patient can get around locally by themselves, by one means of transport or another, and not just by walking.

**Q4b. Were they able to travel without assistance before the injury?**

Record whether the person was able to travel independently before injury.

**Skipping**

If the person can travel, assume that they could also do this before the injury (4b = yes) and skip to Q5a (Work).

#### Follow-up questions

If the person is unable to travel, then check capability: *If you need to get somewhere, can you call a taxi or arrange a lift from a friend*? As well as calling the taxi, the person needs to be able to tell the driver where to go, and to behave appropriately and safely when out independently in the community.

**Specific issues**

The person does not need to be able to travel by public transport, such as a bus or underground/subway. This question is not about being able to afford transport (e.g., taxis), but about the tasks involved. Sometimes particular circumstances make local travel difficult, and the question can be put hypothetically.

### Work

**Q5a. Are they currently able to work to their previous capacity?**

Work refers to jobs that are paid at a reasonable rate, which in principle at least, are open to others (“competitive”). “Non-competitive work” includes work done voluntarily, jobs that are specifically designated for disabled people, and work in sheltered workshops. Other roles taken as equivalent to “work” here are studying as a student and being a caregiver.

**Q5b. How restricted are they?**

a) Reduced work capacity.
b) Able to work only in a sheltered workshop or non-competitive job, or currently unable to work.

If the person is not able to work to their previous capacity, record the level of restriction. Any of the following indicate reduced capacity for work: 1) change in level of skill or responsibility required; 2) change from full-time to part-time working; 3) special arrangements made by an employer (e.g., increased supervision at work); and (d) change from steady to casual employment (i.e., no longer able to hold steady job).

Students should be able to return to their previous course, and not have noted changes in their ability to study. If someone has been absent from school because of injury, then disruption of studies caused by the absence itself should be discounted. Examples of problems that indicate reduced capacity for study are: 1) increased difficulty in studying (needing to spend more time than before to keep up); 2) unaccustomed problems with progress (e.g., failing examinations); and 3) revised program of study because of problems (e.g., studying for a lesser qualification).

**Students Q5b.** (a) If the student has a reduced capacity for study but is still studying, then they are Upper MD; and (b) if the student is currently unable to study, then they are Lower MD.

**Q5c. Were they either working or seeking employment before the injury (answer “yes”) or were they doing neither (answer “no”)?**

Confirm whether the person was working/looking for work, or was a student, or a caregiver before injury. If they did none of these, then record “no.”

**Skipping**

If the person is able to work at their previous capacity, then assume that they were working before injury (5c = yes) and skip Question 5b.

Work is only used in the rating if the person was in a work role before injury or looking for work. If not (e.g., retired), then skip Questions 5a and 5b and check “No” at 5c to indicate that they did not participate pre-injury.

#### Follow-up questions

If the person has returned to work, check to see whether there are any changes in their hours or what they are able to do. Has another person taken on some of their previous responsibilities?

If the person reports a change, ask why this has happened. Sometimes change in employment status may be unrelated to injury (e.g., because of end of contract or redundancy/layoff). Such changes do not indicate a reduced capacity for work. Lack of local opportunities for employment is also a factor that needs to be discounted.

If the person was not working before, but was available for work, they can still be assessed, but the questions are hypothetical. You can ask questions such as: *Do you think you would be able to work at the same capacity as before?; Do you think there would be problems related to the injury that would cause difficulty in your ability to work to your previous capacity*?

**Specific issues**

- Many elderly patients will have retired, and this section will not be relevant. Social and leisure activities then become particularly important in establishing a rating.
- The respondent's view of what constitutes change from full time to part time is usually accepted, given that it will vary from job to job. If there is uncertainty, a guide to assess is a reduction to ≤30 h in a job which is usually 35 h per week; or, more generally for other work patterns, a reduction of more than 10% from full-time. Thus, small adjustments in work patterns (e.g., by half a day per week) would not be counted, while more than this would be considered a restriction. The focus is on function at the current time point. Future plans, such as going back to work next week, are not included.
- Usually if a person is on sick leave as judged by a doctor, they are recorded as unable to work, even if they think they could work. However, sometimes there is a policy of putting people on leave irrespective of their actual ability to work (e.g., in some systems people may automatically be put on leave for several months after a neurosurgical operation). In such cases, a judgment must be made about whether the person is capable of working. For example, if the person reports that they are capable of working and has few or no symptoms, then it is reasonable to rate them as able to work.
- A patient may be prevented from doing certain kinds of work activity (e.g., driving) because there is a risk of post-traumatic epilepsy, although the person has not actually had a seizure. In these cases, the restriction should be ignored as far as possible for the purposes of rating. On the other hand, if the patient has actually suffered a seizure, then limitations imposed by the risk of epilepsy should be taken into account.
- Judging whether someone is capable of working when they are not actually working can be difficult because problems may only become apparent when the person actually returns to work. In this case, asking about symptoms and problems that might interfere with work can help to inform the judgment. Normally, ability to work is indicative of independence; however, occasionally, someone in the Upper SD range may be working in sheltered employment.
- In cases where the person performed more than one major role before injury (e.g., working and caregiving), these can be treated together when considering change in status.

### Students

For students, the work questions are adapted as follows: *Are you able to return to perform your school/college work as well as before? Are you attending for the same number of hours as before your injury? Are you studying the same number of subjects*?

### Caregivers

Taking care of others can also be considered equivalent to work, provided it was a major role before injury. Caregiving is a major role if someone else needs to be found to take on these particular responsibilities if the patient is no longer able to perform these. This can be taking care of children, taking care of someone else's children during the day (e.g., looking after a relative's children so that the relative can work), or care for a dependent relative.

### Social and leisure activities

**Q6a. Are they able to resume regular social and leisure activities outside home?**

Social and leisure activities vary depending on the individual and can be any specific free-time activities which the person does for pleasure and recreation.

**Q6b. What is the extent of restriction on their social and leisure activities?**

a) Participate a bit less: at least half as often as before injury.
b) Participate much less: less than half as often.
c) Unable to participate: rarely, if ever, take part.

If the person is unable to resume previous social and leisure activities, then record the amount of restriction.

**Skipping**

If they are able to resume social and leisure activities, then assume that they participated before injury (6c = yes), skip 6b, and go to 7a.

If it is not possible to establish any regular pre-injury social and leisure activities, skip Questions 6a and 6b and check “No” for Question 6c.

#### Follow-up questions

Start by asking about the person's main activities before injury, and then ask about what they do now. Probe with specific questions: *How did you spend your day before the injury? How often did you get out? What activities did you do in your free time? Do you think your level of activity has changed*?

Patients can be prompted with suggestions such as: 1) sport, for example, football and swimming; 2) going to sporting events as a spectator; 3) walking; 4) going to a club or pub; and 5) visiting friends. If it proves difficult to find activities outside the home, then consider the full range of activities, including those at home, such as gardening, reading, video games, browsing the Web, social media, etc.

If the person reports a change, then ask why this has happened. People may be temporarily restricted by circumstances from engaging in their usual leisure activities. For example, change in financial circumstances may produce a change in social activities, but this is not relevant. Some leisure activities are seasonal, etc. On the other hand, typical brain injury problems that may interfere with social and leisure activities are: lack of motivation or initiative; avoidance of social involvement; physical problems such as loss of mobility; cognitive problems such as poor concentration; and problems such as poor temper control or impatience.

**Q6b. What is the extent of restriction on their social and leisure activities?**

Ask the person how often they participated in activities before the injury (i.e., how many occasions per week) and how often they participate now.

**Q6c. Did they engage in regular social and leisure activities outside home before the injury?**

Almost everyone will have some regular pre-injury social and leisure activities, but it may take prompting to elicit these.

**Specific issues**

- Sometimes people misunderstand what is meant by this question, and it needs to be explained that any free-time activity is relevant. The priority here is to identify change in activities outside the home, but if the person engaged in little or no activity outside the home before injury, then activities at home can be included.
- “Outside home” here means beyond the person's private space (with private space potentially including one's garden or yard).
- Rating people in rehabilitation or in some other institutional care is difficult because the person has not had the opportunity to resume normal social and leisure activities. Usually, someone in care will be rated as dependent, and the social and leisure activities section can then be omitted. However, if the person is independent, then an effort should be made to rate them on the basis of what they expect they would be able to do if they were living at home.
- Measuring extent of participation in terms of occasions per week emphasizes a quantifiable aspect of social and leisure activities. Sometimes, quality of participation is affected by brain injury; for example, the person may become a spectator in a sport rather than an active participant. However, changes such as this are very difficult to quantify and can reflect the especially demanding nature of some sports. Thus, for the sake of simplicity, it is the fact of participation that is rated in the interview. One of the main consequences of brain injury is withdrawal from activities involving social interaction, and the simple approach here is sensitive to such changes.
- Social and leisure activities will vary depending on the age and background of the participant. Considering activities at home is particularly relevant in older age groups.
- Assessing people who had problems of alcohol or drug dependence before injury can be problematic, given that pre-injury activities may have revolved around their dependence. It is acceptable to use judgment in these cases. Sometimes it may not be sensible to complete the section on social and leisure activities as it stands, but one would still consider overall change in function when considering the rating on the GOSE. In principle, one asks about activities before injury and activities now, and whether these have changed.

### Family and friendships

**Q7a. Have there been psychological problems which have resulted in ongoing family disruption or disruption to friendships?**

The question is directed at assessing alterations in close relationships as a result of injury. Changes may be increased friction in relationships, but also can take the form of withdrawal or isolation.

**Skipping**

If the person claims no problems, assume that they did not have problems before injury (7c = no) and skip Question 7b.

#### Follow-up questions

A list of typical post–brain injury changes is given in order to help elicit problems in relationships, an area in which the person may be reluctant to admit problems. It can be useful to go through the problems listed, particularly change in mood, because these are likely to affect relationships. This question is not intended to find out whether they are irritable, etc.—these are examples of changes that can impact relationships. Relationship problems may arise from other sources, such as cognitive impairment or injury-related physical impairment, and these are also counted as sources of disruption. The aim is to establish whether their relationships are strained, and if so, how much.

**Q7b. What has been the extent of disruption or strain?**

The following definitions apply: 1) Occasional—some problems post-injury, but less than once a week and not causing continuous strain. For example, occasional bad temper, but things blow over; 2) Frequent—problems at least weekly, strain on relationships, but regarded as tolerable. For example, temper outbursts at least once a week resulting in modification of closeness of relationships; and 3) Constant daily problems—breakdown or threatened breakdown of relationship within family or friendship; problems regarded as intolerable. If the patient has become very withdrawn and socially isolated as a result of injury, then this also represents constant disruption.

**Q7c. Were there similar problems with family or friends before the injury?**

The question concerns whether similar problems were present before injury. Confirm that any problems with relationships are new since the injury, or at least have become markedly worse since the injury. If the person says that similar problems were present before injury, then a follow-up question must be asked to establish whether things are significantly worse. If they are worse, Question 7c should be marked “No.”

**Specific issues**

The presence of a reported change in personality or other post-TBI impairment is not of itself sufficient to warrant classifying the person as moderately disabled—the change must be having an adverse impact on family and friendships.

This question should consider/elicit whether a patient has become isolated and/or withdrawn since their injury. In this case, it is more relevant to consider how tolerable this is for others rather than the frequency of the problem.

Impact on relationships with others is an area in which people may lack insight, and it can be very useful to ask a surrogate about this aspect of life.

### Return to normal life

**Q8a. Are there any other current problems relating to the injury which affect daily life?**

The question concerns symptoms that have arisen since the injury that are significant enough to impinge on functioning in everyday life.

**Skipping**

If the person reports that they do not have any problems, assume that they did not have any problems before injury (8b = no).

#### Follow-up questions

The symptom list on the interview schedule can be used: *Do you have any of the following problems from the injury: headaches, dizziness, tiredness, sensitivity to noise or light, slowness, memory failures, and concentration problems*? The list of problems here includes those reported for post-concussion syndrome.

Confirm any symptoms that impact daily life: Do you find that that (symptom) has an effect on what you do in daily life?

Examples include: A movement problem that interferes with tasks involving fine motor control (such as shaving) would be counted, while a change that the person describes as “not bothersome” would not.

**Q8b. Were similar problems present before the injury?**

Confirm that any problems or symptoms reported are new since the injury, or at least have become significantly worse after the injury. When you ask whether these problems were present before and the answer is “yes” (to headaches for example); if these headaches are worse now or more frequent, mark the pre-injury question 8b as a “no.”

**Specific issues**

Similar problems are reported in the general population. It is thus important to establish that the problems have developed since the injury, and to exclude common problems and complaints that were present before injury.

Sometimes people report minor issues, and judgment should be applied as to what counts.

It is not necessary to be exhaustive in identifying symptoms.

The symptoms listed are not specific to brain injury and can arise from other causes, such as depression. If the person gives some other explanation, for example, bereavement, or anxiety over a pending court case, then the symptoms are not counted in the rating. However, the assessor is not expected to make a detailed investigation of this point or make a fine judgment as to cause. If there is any doubt, symptoms arising after the injury should be included in the rating.

## Special Issues for Assessment

### Rating people who were severely disabled before injury

Patients who were dependent before injury represent a challenge for rating because the effects of injury on function are often difficult to identify unambiguously. The original description of the GOSE structured interview proposed that such patients should simply be identified as a separate category.^8^ However, in practice it is often helpful if patients can also be assimilated into the standard scale for analysis.

In such cases, assessors are asked to make a judgment concerning the appropriate rating on the scale. In keeping with the general principles behind the GOSE, this should focus on changes that have taken place after injury. Thus, for example, a person who reports no problems or symptoms would be rated as Upper GR, whereas a person who reports that their dependence has increased significantly would be rated as Upper or Lower SD. It is understood that this rating requires judgment, but it is in keeping with the way that the GOS has traditionally been applied.

### Rating people who are in hospital or care

The GOSE is not intended for use during acute hospitilization after TBI, but patients who are in hospital or care at follow-up can potentially be assessed.

It is often obvious from the level of assistance needed that patients who are in the hospital or some other form of care are dependent. Such patients will therefore be assigned an outcome of either Upper or Lower SD depending on their level of function in the institution. Given that the person has not had the opportunity to resume life at home, the rest of the interview will often be skipped. These cases are thus an exception to the recommendation to complete the entire interview. In general, we strongly encourage interviewers to administer the entire interview, given that later responses may lead to re-evaluation of earlier answers.

Patients may be in the hospital when independent for a variety of reasons, including social circumstances, medical considerations, or in-patient rehabilitation. Patients who are about to be discharged may well also be independent. In these cases, it is necessary to complete the later parts of the interview, adapting aspects of the sections as necessary. Relevant questions include: Is the person cleared to leave the unit on their own, able to go to the gift shop and make a purchase, and cleared to leave the hospital without supervision? If the person is being discharged, the destination is relevant. Discharge to care at home or to another institution normally indicates continued dependence.

Persons who are detained in prison or a similar institution are usually independent, and the interview will need to be adapted appropriately to fit the circumstances.

### Rating people who have an illness unconnected to the injury

If the person has an illness that is clearly unconnected to the injury, this should be discounted when making a rating: for example, if the person is off of work because of flu when the follow-up is scheduled, or the person has had an operation for an unrelated condition such as a hip replacement. The assessor can ask about the person's level of functional recovery before the illness or operation, and the limitations that they believe are now attributable to illness.

### Rating people who have injuries to other parts of the body (peripheral injuries/systemic illness)

The GOSE rating is based on changes post-injury and, as originally described, does not distinguish between brain injury and other types of injury that occurred at the same time. The GOSE can be used in this way to assess the consequences of all injuries (“GOSE-All”), including polytrauma and any side effects of an intervention. Alternatively, the assessor can choose to focus on the specific effects of TBI (“GOSE-TBI”). The decision about whether to assess GOSE-All or GOSE-TBI will depend on the purpose of the study. The approach taken should be clearly specified.

### Glasgow Outcome Scale-Extended/traumatic brain injury: the primary assessment concerns the impact of brain injury

An effort should be made to discount the effects of peripheral injuries that occurred at the same time as the TBI (e.g., a person with otherwise GR is unable to return to work because of a broken leg). Only *discount* disability that is clearly *unrelated* to brain injury. If there is any doubt, the effects should be included in the rating. Complaints that are hard to separate as to whether they might be attributable to peripheral injuries (such as fatigue, lack of initiative, or depression) would almost always be considered TBI related. We recognize, however, that in practice it may be difficult to disentangle effects of systemic injuries from those of brain injury, and that attempting to do so risks introducing an element of subjectivity.

#### Procedure

As part of the interview, ask the patient whether they have limitations that they believe are attributable to injuries/illness to other parts of the body and not the brain injury. If they have disability attributable to peripheral injuries, ask how they think they would do without those limitations, concentrating on sections that have an impact on the rating. If the person is not sure whether the problem arises from brain injury or something else, count it as attributable to the brain injury.

## Discussion

This Manual was developed as part of the CENTER-TBI and TRACK-TBI studies and has been refined and elaborated upon from the experience in these studies. It is intended to be part of a data-quality management strategy for outcome assessment and to be useful to investigators using the GOSE in single and multi-center studies.

### Training and monitoring

Implementing appropriate strategies for data-quality management is an important part of any project.^17^ Single-center studies are able to address the issue of inter-rater differences by restricting the number of assessors involved and ensuring communication on borderline cases and other issues. The current Manual can provide support to such studies, and if assessors are already experienced, there will be no need for formal training. For multi-center studies in which the GOSE is a primary end-point, maximizing inter-rater agreement is a key issue. Choi and colleagues^18^ showed that variability in outcome assessment affects not only the power of a study, but also the size of the differences that are found. Thus, reducing variability is critical and particularly important for pharmaceutical trials, where expected effect sizes are small. Experience in multi-center studies supports four critical steps for data-quality enhancement:

1. Specification in advance as to whether ratings should reflect disability caused by the consequences of all injuries (i.e., brain and peripheral body parts) or only the specific effects of the brain injury.
2. Initial training that covers the procedures for completing the assessment and includes consideration of how to deal with cases that are borderline or hard to classify.
3. A process for accreditation involving satisfactory completion of assessments, either case vignettes or “live” cases.
4. Central monitoring of assessments for completeness and consistency, including feedback to assessors of issues that arise and the opportunity to review and change assessments.

It is important to decide whether the GOSE-All or GOSE-TBI approach is being used and to communicate this to assessors. The contrast between the two approaches emerged during harmonization of the CENTER-TBI and TRACK-TBI projects and appears to reflect different practices in Europe and North America. Anecdotally, it was known that the “GOSE-All” approach was commonly used in European studies, whereas the “GOSE-TBI” assessment was typical for trials in the United States. However, past clinical trials have rarely specified which approach was being used in the formal report.^19^ This is an important omission. Studies using the GOSE-All approach may wish to include a measure of extracranial injury as a covariate in the analysis.

Steps for improving data quality are particularly important in studies in which the GOSE is a primary end-point, where there is a desire to maximize rigor and minimize variability. For example, in a multi-center trial of dexanabinol in severe brain injury, implementation of training and monitoring were found to reduce queries concerning inconsistencies in assessment from 30% or, more initially, to <10% later in the study.^20^ Similar experience is reported by Boase and colleagues,^13^ who describe in detail GOSE curation in the TRACK-TBI study.

The initial training should include instructions as to how to administer the GOSE and derive an overall score. It is worth emphasizing the issue of scoring, given that it will be unfamiliar to persons accustomed to assessing a GOS in the classic format of an overall judgment made by the clinician. One of the aspects of the assessment that often surprises persons is the amount of disagreement in scoring discovered between assessors. It can be useful to note this as a demonstration vignette early in training to motivate the process. A mistake that inexperienced assessors often make is to complete the interview mechanically without using follow-up questions, and in group training, it is useful to use case studies that are ambiguous and require trainees to identify sections where further information is needed to arrive at a rating. Other useful training elements include observation of “live” interviews and recording the assessor's own interviews for direct feedback. As the study progresses, it is useful if assessors from multiple centers have the opportunity to convene regularly with supervisors/principal investigators to discuss problematic cases and other issues arising with assessment.^13^

### Strengths and limitations

The GOSE is often criticized for being a relatively coarse outcome. Nonetheless, major strengths of the assessment are its ability to assign an outcome in all cases, the fact that categories are clinically meaningful, and that it focuses on changes post-injury. The latter lends it particular sensitivity to brain injury severity. Despite the simplicity of the scale, it has proven to be useful in a wide variety of contexts. In TRACK-TBI, the scale has been found to be useful in mild TBI.^21^ In this study, in addition to the overall GOSE rating, responses recorded in the “Relationships” section were informative as individual outcomes, demonstrating that more can be extracted from the assessment than simply a single score.

The GOSE is intended for assessing groups of cases in an efficient manner, and there are limits to the precision that is achieved on the basis of a short interview. Some of the criticisms of the GOSE are based on a misunderstanding of the intended use of the scale and unrealistic expectations of precision from a brief assessment. As already described, some cases fall readily into one category or another. Borderline cases give rise to uncertainty of one category, and differences of one category can be considered to be within the normal measurement error of the assessment. For some studies, this level of accuracy will be sufficient, while others will seek to maximize precision. Sharpening the assessment takes some effort, including the data-quality management steps already outlined. There will nonetheless be limits to how accurate an assessment based predominantly on patient and collateral reports can be.

In principle, it is possible to extend the rigor of the assessment by using the GOSE as a framework within which other assessments can contribute greater confidence in the final rating. For example, in the province of Ontario, Canada, the GOSE is used to define “catastrophic impairment” after TBI for no-fault compensation.^22^ As codified in legislation, a rating of Upper SD or less at 6 months or Lower MD or less at 12 months are considered to be “catastrophic.” That is, these indicate long-lasting, life-changing levels of disability. Clearly, the award of compensation is not based simply on interviewing the patient, no matter how skillful the assessor. In these cases, a wide variety of information is collected in evidence, and medico-legal expertise has been developed locally in using the GOSE as a framework for deciding eligibility for compensation.^23^ Such an exhaustive process is impractical in the setting of a multi-center study, but it serves to suggest possible ways in which global outcome assessment could be refined by additional assessments.

At the other end of the spectrum, the GOSE can be assessed by a questionnaire that is completed by the patient or a caregiver and then scored centrally. This approach has been used in several recent clinical studies^24–26^ and is particularly useful in situations such as surgical treatment trials, where blinding of assessors is difficult. Interviewing is thus not essential to the assessment, although, done well, should lend extra precision and robustness.

The Manual describes circumstances in which the GOSE can be applied to patients who are in the hospital, but some aspects of the scale are only relevant if patients have returned to the community. Nonetheless, potentially useful information concerning function can be collected before discharge from the hospital in a manner that parallels the GOSE.^27^ The information may contribute to better prediction of outcome on the GOSE.

### Clinimetric properties

The GOSE is an ordinal scale consisting of a hierarchy of discrete categories, and each of the steps from death to complete recovery (return to pre-injury life) is multi-dimensional and global in nature. Thus, there is not a latent unidimensional ability underlying all states described by the scale. For example, although the construct of disability runs through much of the GOSE, it does not apply to lower parts of the scale such as death versus survival. Feinstein^28^ describes similar types of scales, including the Apgar assessment of infant health and indices of socioeconomic status, that are composites of selected indicators. The Glasgow Coma Scale^29^ is another example of such a scale.

Key requirements are that the GOSE should have value in describing clinically important phenomena and that it can be applied consistently by assessors. It is not necessary that items should be correlated. For example, participation in work and participation in social and leisure activities need not be positively correlated. Nonetheless, there are expectations of logical relationships between different parts of the assessment. For example, as described in the Manual, it is inconsistent for someone who reports that they are back at work to also report that they are dependent in daily life. The assessor is expected to identify and reconcile such inconsistencies.

Participation is assessed in relation to pre-injury status, and this means that some aspects of the assessment reflect relative change for the person. For example, someone with high pre-injury vocational demands may find reintegration more difficult than the person with less exacting work. Similarly, patients who were retired before injury may find it easier to return to their normal social and leisure activities and may consequently show better recovery than a younger person who was engaged in full-time work before injury. These relationships have been described in mild TBI by van der Naalt and colleagues,^30^ who showed that in more highly educated persons, there may be a U-shaped relationship between outcome and aging. The principle here is that return to normal life will depend, in part, on how demanding participation in activities was before injury. The GOSE differs in this respect from health outcomes, such as for example, the 36-Item Short Form Survey, that are norm based.

### Approaches to analysis

In clinical trials the GOS and GOSE are often dichotomized in analysis into “favorable” and “unfavorable” outcomes. This yields a clear interpretation of findings, but can be criticized because it reduces the scales to two outcomes. Ordinal analysis has been shown to have greater statistical power than simple dichotomization^31,32^ and therefore is preferred for many purposes. Adjustment for baseline covariates can also improve power to detect effects on the GOS and GOSE, and there is an extensive literature on TBI outcome prediction that can inform selection of covariates.^33^ Cumulative link models, and specifically the proportional odds model, a form of ordinal logistic regression, have been widely used in work on TBI prognosis. Other ordinal analysis strategies include the sliding approach to dichotomization proposed by Murray and colleagues.^34^ For a sliding dichotomy, the binary outcome is tailored to the baseline prognosis of the person.

These methods measure shift of persons over a range of outcomes and provide a useful summary of overall relationships. In some contexts, for example genetics, the focus may be on differences between categories. In this case, a partial proportional odds approach or an all-nominal cumulative link model may be used to examine the association of genomic data with each possible GOSE dichotomization. This latter approach to analysis is consistent with conceptualizing the GOSE as consisting of discrete categories which may have their own specific associations with other factors.

## Conclusion

The GOSE has become the most frequently adopted measure of global outcome in TBI clinical trials and research studies. It is used internationally and is the preferred primary outcome of efficacy for FDA-regulated drug and device trials. Maximizing the ability to assign GOSE scores uniformly within and across single- and multi-center studies is thus of key importance. We hope that this Manual for the GOSE, aimed at assessors, will contribute to facilitating agreement and support the ongoing use of the scale.

## Supplementary Material

Supplemental data
