# Supplementary material for: A Manual for the Glasgow Outcome Scale-Extended Interview
Source: J Neurotrauma. 2021 Aug 13;38(17):2435–46. doi: 10.1089/neu.2020.7527 (PMC8390784; doi:10.1089/neu.2020.7527)
Supplement: Supplemental data [file Supp_DataSA.docx]

**Appendix 1 Glasgow Outcome Scale - Extended Interview: Schedule and Scoring Sheet**

**Glasgow Outcome Scale - Extended**

Patient's name: ___________________________________ Date of interview: ___________

Date of Birth: _____________ Date of injury ______________ Gender: M / F

Age at injury: ___________ Interval post-injury: _____________

Respondent: Patient alone ___ Relative/ friend/ carer alone ___ Patient + relative/ friend/ carer ___

Interviewer: ______________________________

|  | CONSCIOUSNESS |  |  |  |  | | |
| --- | --- | --- | --- | --- | --- | --- | --- |
| 1. | Is the head injured person able to obey simple commands, or say any words? |  |  |  | 1 = No (VS)  2 = Yes | | |
|  |  |  |  |  |  | | |
| Anyone who shows ability to obey even simple commands, or utter any word or communicate specifically in any other way is no longer considered to be in the vegetative state. Eye movements are not reliable evidence of meaningful responsiveness. Corroborate with nursing staff. Confirmation of VS requires full assessment as in the Royal College of Physician Guidelines. | | | | | | | |
|  |  |  |  |  |  | | |
|  |  |  |  |  |  | | |
|  | INDEPENDENCE IN THE HOME |  |  |  |  | | |
| 2a | Is the assistance of another person at home essential every day for some activities of daily living? |  |  |  | 1 = No  2 = Yes | If “No” go to 3a. | |
|  |  |  |  |  |  | | |
| For a ‘No’ answer they should be able to look after themselves at home for 24 hours if necessary, though they need not actually look after themselves. Independence includes the ability to plan for and carry out the following activities: getting washed, putting on clean clothes without prompting, preparing food for themselves, dealing with callers, and handling minor domestic crises. The person should be able to carry out activities without needing prompting or reminding, and should be capable of being left alone overnight. | | | | | | | |
|  |  |  |  |  |  | | |
| 2b | Do they need frequent help or someone to be around at home most of the time? |  |  |  | 1 = No (Upper SD)  2 = Yes (Lower SD) | | |
|  |  |  |  |  |  | | |
| For a ‘No’ answer they should be able to look after themselves at home for up to 8 hours during the day if necessary, though they need not actually look after themselves. | | | | | | | |
|  |  |  |  |  |  | | |
| 2c | Was assistance at home essential before the injury? |  |  |  | 1 = No  2 = Yes | | |
|  |  |  |  |  |  | | |
|  | INDEPENDENCE OUTSIDE THE HOME |  |  |  |  | | |
| 3a | Are they able to shop without assistance? |  |  |  | 1 = No (Upper SD)  2 = Yes If ‘Yes’ go to 4a | | |
|  |  |  |  |  |  | | |
| This includes being able to plan what to buy, take care of money themselves, and behave appropriately in public. They need not normally shop, but must be able to do so. | | | | | | | |
|  |  |  |  |  |  | | |
| 3b | Were they able to shop without assistance before the injury? |  |  |  | 1 = No  2 = Yes | | |
|  |  |  |  |  |  | | |
|  |  |  |  |  |  | | |
| 4a | Are they able to travel locally without assistance? |  |  |  | 1 = No (Upper SD)  2 = Yes If ‘Yes’ go to 5a | | |
|  |  |  |  |  |  | | |
| They may drive or use public transport to get around. Ability to use a taxi is sufficient, provided the person can phone for it themselves and instruct the driver. | | | | | | | |
|  |  |  |  |  |  | | |
| 4b | Were they able to travel without assistance before the injury? |  |  |  | 1 = No  2 = Yes | | |
|  |  |  |  |  |  | | |
|  |  |  |  |  |  | | |
|  | WORK |  |  |  |  | | |
| 5a | Are they currently able to work to their previous capacity? |  |  |  | 1 = No  2 = Yes | If ‘Yes’ go to 6a | |
|  |  |  |  |  |  | | |
| If they were working before, then their current capacity for work should be at the same level. If they were seeking work before, then the injury should not have adversely affected their chances of obtaining work or the level of work for which they are eligible. If the patient was a student before injury then their capacity for study should not have been adversely affected. | | | | | | | |
|  |  |  |  |  |  | | |
| 5b | How restricted are they?   1. Reduced work capacity. |  |  |  | 1 = a (Upper MD) | | |
|  | b) Able to work only in a sheltered workshop or non-competitive job, or currently unable to work. |  |  |  | 2 = b (Lower MD) | | |
|  |  |  |  |  |  | | |
| 5c | Were they either working or seeking employment before the injury (answer ‘yes’) or were they doing neither (answer ‘no’)? |  |  |  | 1 = No  2 = Yes | | |
|  |  |  |  |  |  | | |
|  | SOCIAL & LEISURE ACTIVITIES |  |  |  |  | | |
| 6a | Are they able to resume regular social and leisure activities outside home? |  |  |  | 1 = No  2 = Yes | If ‘Yes’ go to 7a | |
|  |  |  |  |  |  | | |
| They need not have resumed all their previous leisure activities, but should not be prevented by physical or mental impairment. If they have stopped the majority of activities because of loss of interest or motivation then this is also considered a disability. | | | | | | | |
|  |  |  |  |  |  | | |
| 6b | What is the extent of restriction on their social and leisure activities?   1. Participate a bit less: at least half as often as before injury. |  |  |  | 1 = a (Lower GR) | | |
|  | 1. Participate much less: less than half as often. 2. Unable to participate: rarely, if ever, take part. |  |  |  | 2 = b (Upper MD)  3 = c (Lower MD) | | |
|  |  |  |  |  |  | | |
| 6c | Did they engage in regular social and leisure activities outside home before the injury? |  |  |  | 1 = No  2 = Yes | | |
|  |  |  |  |  |  | | |
|  | FAMILY & FRIENDSHIPS |  |  |  |  | | |
| 7a | Have there been psychological problems which have resulted in ongoing family disruption or disruption to friendships? |  |  |  | 1 = No  2 = Yes | If ‘No’ go to 8a | |
|  |  |  |  |  |  | | |
| Typical post-traumatic personality changes: quick temper, irritability, anxiety, insensitivity to others, mood swings, depression, and unreasonable or childish behaviour. | | | | | | | |
|  |  |  |  |  |  | | |
| 7b | What has been the extent of disruption or strain?   1. Occasional - less than weekly |  |  |  | 1 = a (Lower GR) | | |
|  | 1. Frequent - once a week or more, but tolerable. 2. Constant - daily and intolerable. |  |  |  | 2 = b (Upper MD)  3 = c (Lower MD) | | |
|  |  |  |  |  |  | | |
| 7c | Were there problems with family or friends before the injury? |  |  |  | 1 = No  2 = Yes | | |
| If there were some problems before injury, but these have become markedly worse since injury then answer ‘No’ to 7c. | | | | | | | |
|  |  |  |  |  |  | | |
|  | RETURN TO NORMAL LIFE |  |  |  |  | | |
| 8a | Are there any other current problems relating to the injury which affect daily life? |  |  |  | 1 = No (Upper GR)  2 = Yes (Lower GR) | | If ‘No’ go to  next page |
|  |  |  |  |  |  | | |
| Other typical problems reported after head injury: headaches, dizziness, tiredness, sensitivity to noise or light, slowness, memory failures, and concentration problems. | | | | | | | |
|  |  |  |  |  |  | | |
| 8b | Were similar problems present before the injury? |  |  |  | 1 = No  2 = Yes | | |
| If there were some problems before injury, but these have become markedly worse since injury then answer ‘No’ to 8b. | | | | | | | |

Epilepsy:

Since the injury has the head injured person had any epileptic fits? No / Yes

Have they been told that they are currently at risk of developing epilepsy? No / Yes

What is the most important factor in outcome?

Effects ofhead injury ___ Effects of illness or injury to another part of the body ___ A mixture of these ___

Scoring: The patient’s overall rating is based on the lowest outcome category indicated on the scale. Refer to Guidelines for further information concerning administration and scoring

| 1 | Dead |  |  |  |
| --- | --- | --- | --- | --- |
| 2 | Vegetative State (VS) |  |  |  |
| 3 | Lower Severe Disability (Lower SD) |  |  |  |
| 4 | Upper Severe Disability (Upper SD) |  |  |  |
| 5 | Lower Moderate Disability (Lower MD) |  |  |  |
| 6 | Upper Moderate Disability (Upper MD) |  |  |  |
| 7 | Lower Good Recovery (Lower GR) |  |  |  |
| 8 | Upper Good Recovery (Upper GR) |  | © Lindsay Wilson, Laura Pettigrew, Graham Teasdale 1998 | |
